# Critical Evaluation and Validation of a High-Throughput Microplate-Based Cupric Reducing Antioxidant Capacity Method for the Analysis of Fish Feed Ingredients

**DOI:** 10.3390/antiox14060728

**Published:** 2025-06-14

**Authors:** Aleksander Arnø, Viviana Sarmiento, Odd Elvebø, Pedro Araujo

**Affiliations:** 1 Feed and Nutrition Group, Institute of Marine Research, P.O. Box 1870 Nordnes, N-5817 Bergen, Norway; aleksander.arno@kleppnett.no; 2 Department of Biological Sciences, University of Bergen, Thormøhlens Gate 53A, N-5006 Bergen, Norway; 3 Artic Feed Ingredients, Skolegata 22, 7713 Steinkjer, Norway; viviana.sarmiento@arcticfeed.no (V.S.); odd.elvebo@arcticfeed.no (O.E.)

**Keywords:** cupric ion reducing antioxidant capacity assay, CUPRAC, fish feed ingredients

## Abstract

The cupric ion reducing antioxidant capacity (CUPRAC) assay, originally developed to measure the antioxidant capacity of nutritional products spectrophotometrically, utilized water as the solvent for Trolox. Due to the limited solubility of Trolox in aqueous solutions, the optimization of the solvent system was investigated to enhance analytical performance. Solvent combinations consisting of methanol, ethanol, and water were evaluated to identify the mixture that ensures complete dissolution and maximum absorbance signal, using a ternary plot diagram and mathematical modeling. A methanol/water ratio of 0.64:0.36 was identified as the optimal solvent composition. Under these conditions, the CUPRAC assay demonstrated a linear range of 0–50 μM, a limit of detection of 0.91 μM, and a limit of quantification of 2.75 μM. Precision, expressed as the coefficient of variation, was below 5%, and accuracy—defined as the deviation between nominal and back-calculated concentrations—remained within ±7.0%, in accordance with the variation range recommended by the International Committee on Harmonization. The estimated molar absorption coefficient at the optimized solvent ratio (ε_Trolox_ = 2.62 × 10^4^ L mol^−1^ cm^−1^) was applied to determine the antioxidant capacity of fish commercial feed ingredients containing a mixture of rosemary and olive extracts.

## 1. Introduction

An antioxidant is a chemical compound that can decrease oxidation reactions in a biological system [1,2]. The categorization of an antioxidant as effective or ineffective relies on the speed of its oxidation. Hence, the best antioxidant is oxidized much more quickly than other biomolecules [3]. In a dietary product, the main role of an antioxidant is to decrease oxidation processes to prolong storage and shelf-life [2].

A recent article has indicated that 8000 polyphenols compounds have been reported [4], highlighting the importance of estimating the antioxidant capacity of plants or food products to objectively judge their degrees of protection against detrimental oxidation processes. Antioxidant capacity is a parameter that measures the cumulative action of all antioxidants present in an analytical sample during a set amount of time; therefore, it quantifies the amount of oxidant species reduced by the antioxidants [5]. It is important to note that antioxidant activity is a related but distinct parameter from antioxidant capacity. Antioxidant activity is a kinetic approach that describes the rate at which an antioxidant reacts with reactive species, whereas antioxidant capacity refers to the total amount of reactive species that an antioxidant can neutralize.

The three primary approaches used to measure antioxidant capacity are hydrogen atom transfer (HAT), single electron transfer (SET), and combined HAT-SET methods. Table 1 provides a general overview of the most widely cited antioxidant capacity assays, highlighting their mechanisms, underlying principles, detection methods, and corresponding wavelengths. These approaches are based on radical deactivation by an antioxidant. HAT-based assays measure the antioxidant’s ability to quench the radical by means of the transfer of a hydrogen atom. A well-known example of this kind of approach is the oxygen radical antioxidant capacity (ORAC) assay, which measures the oxidative degradation/damage of a fluorescent molecule (e.g., fluorescein) by a peroxyl free radical. SET-based assays measure the ability of the antioxidant to transfer an electron to reduce the radical. Folin–Ciocalteu, ferric reducing antioxidant power (FRAP), and cupric ion reducing antioxidant capacity (CUPRAC) are the main SET-based assays reported. These specific assays monitor the absorption of the redox reactions at 765, 593, and 450 nm by using a mixture of phosphomolybdate and phosphotungstate (aka Folin–Ciocalteu reagent), a ferric 2,4,6-tripyridyl-s-triazine solution, and a mixture of Cu(II) and neocuproine, to oxidize the antioxidant compounds in the sample, respectively. 2,2-diphenyl-1-picrylhydrazyl (DPPH) and 2,2′-azino-bis(3-ethylbenzthiazoline-6-sulfonic acid) (ABTS) assays are reported to measure both HAT and SET depending on the solvent [6,7]. The former assay measures the capacity of the antioxidants in the sample to reduce the absorption of the radical DPPH* at 515 nm, while the latter assay is based on the scavenging capacity of an antioxidant towards the ABTS^•+^ radical, which has an intense blue-green color that is lost upon reduction to ABTS by the antioxidants in the sample.

**Figure 1:**
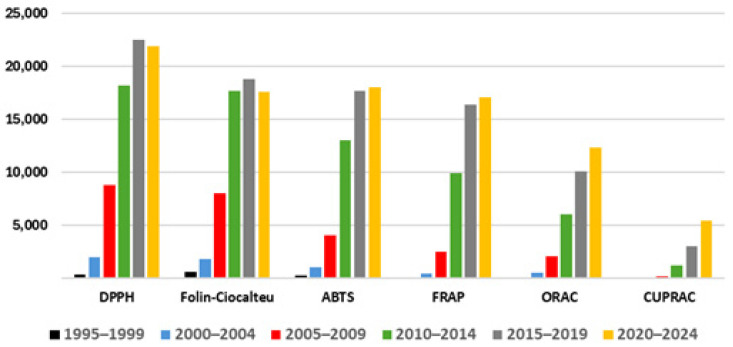
Comparison of different antioxidant capacity assays based on the number of citations reported in Google Scholar and organized in intervals of five years.

A comparative study of the DPPH, Folin–Ciocalteu, ABTS, FRAP, ORAC, and CUPRAC assays in terms of number of citations, cost per 200 samples, and analysis time was conducted using Google Scholar and using the names of the assays and the term polyphenols as keywords. The cost in material to run 200 samples was used to estimate the analysis price based on the prices listed in the Merck catalog (https://www.sigmaaldrich.com). The number 200 was chosen because this the number of analyses that can be run with a fixed amount of limiting reagent from the most economical assay (ABTS). The analysis time was estimated from articles where the different methods are compared [8,9]. The total time was defined as the summation of the different experimental steps where the parameter time is referred to, for instance, incubation, vortex-mixing, or centrifugation times. Unlisted times, such as time spent weighing, pipetting, or diluting, were not considered. The chronological comparison of the different assays for determining the antioxidant capacity revealed that the cumulative number of citations in decreasing order is DPPH > Folin–Ciocalteu > ABTS > FRAP > ORAC > CUPRAC (Figure 1).

The popularity of DPPH has been ascribed to the fact that the oxidant radical DPPH* required for the assay is commercially available [7,10]. A comparison between the periods 2010–2014 and 2015–2019 (Figure 1) revealed that CUPRAC has experienced the highest increase in citations (148%), followed by ORAC (67%), FRAP (66%) ABTS (36%), DPPH (24%), and Folin–Ciocalteu (6%). In addition, the last period (2020–2024) in Figure 1 shows a decline in citations of 6 and 3% for Folin–Ciocalteu and DPPH when compared with the anterior period (2015–2019), respectively, while the rest of the assays revealed an increase in citation ranging from slight (e.g., 2% ABTS and 4% FRAP) to remarkable (e.g., 22% ORAC and 81% CUPRAC).

**Figure 2:**
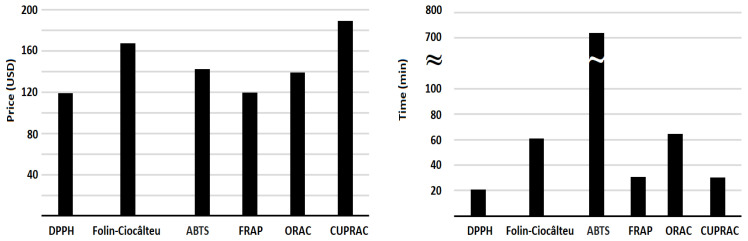
Comparison of different antioxidant capacity assays based on price (USD) and analysis time (minutes).

Generally, there is an inverse relationship between price and demand. As seen in Figure 1, the term demand may be equated with a number of citations, which could explain the observed prices in Figure 2, where DPPH (119 USD) and CUPRAC (189 USD) were the cheapest (most cited) and the most expensive (less cited) assays, respectively. Comparable prices were found for the pairs DPPH/FRAP, ABTS/ORAC, and Folin–Ciocalteu/CUPRAC. Overall, the prices can be regarded as fair values.

The comparison in terms of analysis time revealed that ABTS is the lengthiest assay (Figure 2) due to the long incubation period (720 min) required to produce the radical ABTS^•+^ from ABTS. The remaining methods exhibited reasonable analysis times (~20–65 min) for the determination of the antioxidant capacity of a sample.

The results of the preliminary literature review allow us to rule out (i) ABTS, on the grounds of lengthy preparation time (12 h); (ii) Folin–Ciocalteu and FRAP, as they require precise knowledge of the illumination volume for an accurate measurement of the diffusion coefficient, which makes absolute quantification difficult [11]; (iii) DPPH, as it suffers from pigment interference (e.g., astaxanthin commonly present in salmon feed) [12,13,14]; and (iv) ORAC, on the grounds that measuring fluorescence may not be as common as measuring absorbance. The CUPRAC assay is the least reported method, which could indicate that certain aspects of the assay are understudied. For instance, although the antioxidant reagent (Trolox) is poorly soluble in water, the originally published CUPRAC assay recommends preparing an aqueous solution of Trolox to construct a calibration curve and determine its molar absorption coefficient (ε_Trolox_), without using replicates [9]. On the one hand, it is surprising that the original reference is widely acknowledged as a working assay in different publications, without recognizing the poor solubility of the antioxidant reagent in water [9,15,16,17,18,19,20]. On the other hand, some authors have indirectly reported the lack of solubility of Trolox in water. For instance, a recent publication describes preparing the blank solution (without Trolox) in pure ethanol for calibration [21]. The absence of experimental replicates, statistical parameters (e.g., standard deviations in figures and tables), or statistical analysis is another weakness of the originally published CUPRAC assay. Regardless of the previous observations, it is undeniable that CUPRAC’s popularity has increased gradually, possibly because it allows both hydro- and lipophilic antioxidants to be determined.

Previous validation studies have typically used aqueous solutions of Trolox to determine ε_Trolox_, a critical parameter for accurately assessing the antioxidant capacity of various samples. However, the original CUPRAC publication [9] contains a methodological inconsistency regarding solvent selection for dissolving Trolox. The aim of this research is to address and correct this inconsistency by identifying an optimal solvent composition for Trolox and validating a CUPRAC assay that yields a reliable ε_Trolox_ value. Notably, this study represents the first comprehensive report on the mathematical modeling and validation of a high-throughput, microplate-based CUPRAC assay for determining the antioxidant capacity of fish feed ingredients.

## 2. Materials and Methods

### 2.1. Fish Feed Ingredient Samples

Three commercial feed ingredients (designated as A, B, and C) containing a mixture of rosemary and olive extracts were supplied by Arctic Feed Ingredients (AFI, Brønnøysund, Norway). Both rosemary and olive extracts are rich in antioxidant phenolic compounds like carnosic acid, carnosol, and hydroxytyrosol. The three samples of feed ingredients (A, B, and C) were previously analyzed by liquid chromatography–tandem mass spectrometry to confirm the presence of the mentioned phenolic compounds and the results documented previously [22]. All samples were stored in the dark at room temperature until analysis.

### 2.2. Chemicals and Reagents

Methanol Optima (LCMS grade) and ethanol (HPLC grade) were purchased from Fisher Chemical (Oslo, Norway). Ammonium acetate (reagent grade, ≥98%), copper (II) chloride dihydrate (≥99.99%), neocuproine hydrochloride monohydrate (≥98%), and Trolox (6-hydroxy-2,5,7,8-tetramethylchromane-2-carboxylic acid) were obtained from Merck (Darmstadt, Germany). A Millipore Milli-Q system was used to produce ultrapure water 18 MΩ (Millipore, Milford, CT, USA).

### 2.3. Preparation of CUPRAC Solutions

The solutions required for performing the CUPRAC assay were prepared as follows: 8.52 mg of CuCl_2_·2H_2_O was dissolved in 5 mL of water (10.0 mM); 9.15 mg of neocuproine hydrochloride monohydrate was dissolved in 5 mL of water (7.0 mM); and 7.70 g of ammonium acetate was dissolved in 100 mL of water (1.0 M). An attempt was made to dissolve 3.75 mg of Trolox in 20 mL of water (750 µM), as recommended in the original published assay [9]; however, complete solubility was not achieved. Therefore, the study described in the following section was proposed, which employs a ternary phase diagram to identify the optimal solvent combination that ensures the complete solubility of Trolox.

### 2.4. Selection of the Solvents

A ternary plot diagram was used to identify the optimal solvent combination that maximizes the solubility and absorbance signal (ψ) of Trolox. The solvents tested were methanol (*z*_1_), ethanol (*z*_2_), and water (*z*_3_), with their volume fractions varying between 0 and 1, under the constraint that their sum (*z*_1_ + *z*_2_ + *z*_3_) equals 1. A fixed amount of Trolox (3.75 mg) was used in each test. Seven different solvent mixtures, detailed in Table 2, were evaluated and modeled using the following general equation:

(1) $$\psi =\sum_{i=1}^{i=3}\sum_{j=2}^{j=3}\left[k_{i}\times z_{i}+k_{ij}z_{i}z_{j}\right]$$

In this model, *k_i_* represents the linear coefficients associated with the individual solvents, while *k_ij_* denotes the interaction coefficients between solvent pairs.

### 2.5. Method Validation

The validated performance parameters were those suggested by the International Committee on Harmonization guidelines [23]: more specifically, the analytical range, limit of detection (LOD), limit of quantification (LOQ), precision, and accuracy.

The analytical range was studied by preparing 6 different concentrations of Trolox between 0 and 50 µM, and the relationship signal (y) to concentration (x) expressed as y = φx + β, where the coefficients φ and β represent the slope and intercept of the calibration curve, respectively. The ratio between the standard deviation of a blank solution (σ_blank_) and the slope of the calibration curve (φ) were used to determine the LOD and LOQ as 3.3 × σ_blank_/φ and 10 × σ_blank_/φ, as suggested elsewhere [23,24]. The precision was measured by monitoring the calibration standards in triplicate (5 × 3) over the analysis time (30 min) at 450 nm and 20 °C. The accuracy was estimated by back-calculating the concentrations from the calibration curve and was considered acceptable when its values were within ±15% of the nominal concentration.

### 2.6. Extraction Procedures

The feed ingredient samples (~80 mg) were dissolved in 10 mL of methanol/water (0.64:0.36), vortex-mixed for 20 s (VWR vortex-mixer, Radnor, PA, USA), and centrifuged for 5 min (Centrifuge 5810 R, Eppendorf, Hamburg, Germany) at 1811 g and 20 °C; then, the supernatant was collected and submitted for analysis.

### 2.7. CUPRAC Procedure

The CUPRAC procedure is a modification of the originally published assay [9]. The modification was introduced after selecting the optimal combination of solvents to dissolve Trolox. Briefly, 50 µL of the aqueous solutions CuCl_2_.2H_2_O, neocuproine, and ammonium buffer solutions were consecutively added into a 96-well microplate and incubated at 37 °C for 15 min. After incubation, 100 µL of Trolox or the sample solution, dissolved in a mixture of methanol/water (0.64:0.36), was taken and added to each well, and then submitted for analysis by measuring the absorbance at 450 nm every minute for 30 min. A reagent blank was produced by adding 100 µL methanol/water (0.64:0.36) without Trolox. The Trolox equivalent antioxidant capacity (TEAC in mol/g) was estimated using the expression reported elsewhere [21]:

(2) $$\mathrm{TEAC}=\frac{\Delta A\;\times {\;V}_{f}\times {\;V}_{i}}{\varepsilon_{Trolox}\times {\;V}_{s}\times {\;W}_{s}\times \;h}$$

where the term in the numerator and denominators are the difference in absorbance between the sample and blank (ΔA), the final volume of the assay solution per well (V_f_ = 0.25 mL), the initial volume of the sample (V_i_ = 10 mL), the Trolox molar absorptivity in the CUPRAC reagent (ε*_Trolox_* = 2.62 × 10^4^ L × mol^−1^ × cm^−1^), the volume of the sample used in the assay (V_s_ = 0.10 mL), the weight of the sample (W_s_ = 0.080 g), and the height of the solution in the well (h = 0.50 cm).

### 2.8. UV Spectrophotometry Instrument

A Victor X5 2030 spectrophotometer with a multimode microplate plate reader (Perkin Elmer, Waltham, MA, USA) was used to measure the antioxidant capacity of the samples at 450 nm and 20 °C.

### 2.9. Statistics

The Pearson correlation coefficient (r) and the Fisher ratio that describes the relationship lack-of-fit to pure error variances (aka F-test) were used to judge the validity of the mathematical models derived from the ternary plot diagram and the Trolox calibration. The models were considered linear when the concurrent condition r > 0.990 and F_experimental_ < F_tabulated_ was fulfilled at the 95% confidence level. Statgraphics Centurion XV Version 15.2.11 (StatPoint Technologies, Inc., Warrenton, VA, USA) was used for the comparative analysis of the of fish feed ingredient samples.

## 3. Results and Discussion

### 3.1. Solvent Effect

The results after implementing the ternary plot diagram to select the optimal combination of solvents that yields the maximum solubility and absorption signal (ψ) of Trolox are given in Table 2. In general, the lowest absorption signals were obtained when pure water was used to dissolve Trolox. For instance, the recorded average signals in methanol, ethanol, methanol/ethanol, methanol/water, ethanol/water, and methanol/ethanol/water were 3.07, 2.54, 3.26, 3.29, 2.41, and 2.56 times higher than the average signal in pure water. These results are attributed to the poor solubility of Trolox in water, as confirmed by visually inspecting the 96-well microplate in which Trolox was fully dissolved in all of the pure solvents and combinations described in Table 2, except for the 100% water condition, which exhibited some undissolved powder at the bottom of the wells.

The average standard deviations ($\sigma_{average}$) of the pure (0.039 AU), binary (0.046 AU), and ternary (0.060 AU) systems in Table 2 were used to transform the experimental signals ($\Phi =\psi \times \sigma_{average}$) and model Φ as a function of the variables methanol (*z*_1_), ethanol (*z*_2_), and water (*z*_3_) and their interactions (*z*_1_z_2_, *z*_1_z_3_, and *z*_2_z_3_). The computed model is given by the following expression:

(3) $$\Phi =0.046z_{1}+0.038z_{2}+0.015z_{3}+0.063z_{1}z_{2}+0.112z_{1}z_{3}+0.066z_{2}z_{3}$$

**Figure 3:**
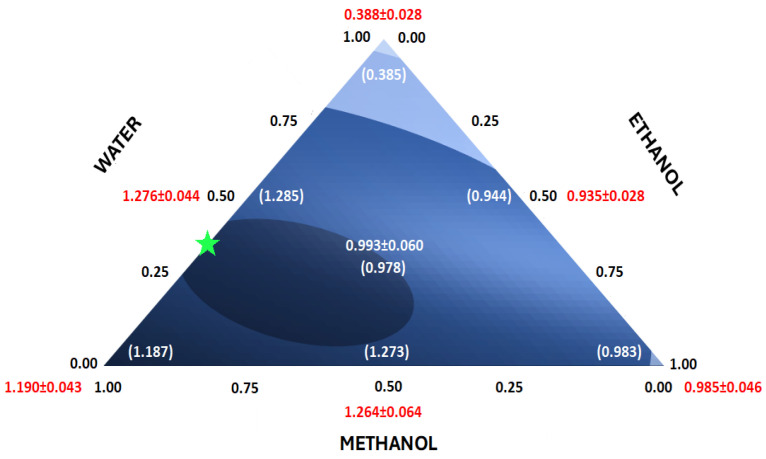
Response surface to visualize the single and combined effect of the solvents used to dissolve Trolox on the UV absorption at 450 nm of the CUPRAC assay. The experimental compositions and signals are described in Table 2 and expressed as means and standard deviations (μ ± σ) in absorbance units (AU). The bracketed numbers inside the triangle are the signals predicted by Equation (3). The green star represents the composition (methanol/water, 0.64:0.36) that yields the maximum absorbance signal (1.332 AU).

Equation (3) predicts that the lowest absorption signal is obtained when pure water is used to dissolve Trolox, and that the interaction of methanol/water has the highest influence on the analytical signal. Its effect is 2.4, 2.9, and 7.5 times higher than pure methanol, ethanol, and water, respectively, and almost two times higher than the binary solvents methanol/ethanol and ethanol/water. The graphical representation of Equation (3) allows the experimental and predicted signals to be visualized and the adequacy of the proposed mathematical model in predicting the absorption signals to be confirmed according to the solvent composition (Figure 3). The best experimental/predicted signals (darkest blue area in Figure 3) were those in the region delimited by methanol (≥0.5) and water (≤0.5). A closer inspection of the darkest blue region indicates that the methanol/water ratio of 0.64:0.36 represents the composition that yields the maximum absorption signal (1.332 AU). This specific composition was selected to validate the CUPRAC assay.

### 3.2. Validation

The originally proposed CUPRAC assay involves the construction of a calibration curve of Trolox in water for the estimation of its molar absorption coefficient (ε_Trolox_ in Equation (2)). Ideally, this parameter is estimated from the slope of the calibration curve (φ) through the expression y = φx = ε_Trolox_βx, where the term β represents the path length. The parameter ε_Trolox_ is influenced by the solvent composition. Therefore, considering the previous results, the linear range for Trolox dissolved in methanol/water (0.64:0.36) was investigated. For this, a five-level curve (0, 50, 100, 200, 300 µM Trolox in triplicate) was examined at 450 nm. This initial evaluation evidenced the lack of linearity between 100 and 300 µM; consequently, the exploration was focused on the lower range of the curve (0–50 µM) by preparing six equally spaced concentration levels of Trolox (0, 10, 20, 30, 40, 50 µM). The recorded signals at the different concentration levels along with the various validation parameters are presented in Table 3. The experimental Pearson correlation coefficient (r = 0.998) and F_experimental_ (3.165) values fulfilled the preestablished condition of r > 0.990 and F_theoretical_ <3.259 at the 95% confidence level, respectively. Therefore, the linearity of the regression model y = 0.013x + 0.039 was confirmed in the range of 0–50 µM of Trolox dissolved in methanol/water (0.64:0.36) with 4 and 12 degrees of freedom for lack-of-fit and pure errors, respectively. The estimated value of ε_Trolox_ from the calibration function (2.62 × 10^4^ L mol^−1^ cm^−1^) was higher than the reported value in water (1.67 × 10^4^ L mol^−1^ cm^−1^) [9], which indicates that there is a high probability that the total antioxidant capacity has been overestimated in those works reporting Trolox calibration curves in pure water. Interestingly, the authors of the originally published CUPRAC method have a patent where the Trolox was dissolved in ethanol and the estimated ε_Trolox_ value (2.40 × 10^4^ L mol^−1^ cm^−1^) at 450 nm [25] was much closer to that reported in the present research.

The LOD and LOQ values, commonly referred as indicators of the sensitivity of analytical methods, were 0.91 and 2.75 µM and similar to previously reported values of 1.01 and 3.33 µM in ethanol at 450 nm, respectively [25]. The precision of the analysis was determined by monitoring the absorbances between 10 and 50 µM Trolox in methanol/water (0.64:0.36), for 30 min at 450 nm, in triplicate (5 × 3 × 30). The coefficient of variation of the signals, expressed as the ratio standard deviation to average (CV = 100 × σ/μ), ranged from 0.6 to 4.3%, indicating that the assay is highly reproducible over time and also accounts for the stability of the standards during the analysis. The accuracy expressed as the percentage of difference between nominal and back-calculated concentration varied around ±7.0% and was regarded as highly accurate considering that it was lower than the maximum ±15% value suggested by the International Committee on Harmonization [23].

### 3.3. Quantitative Analysis of Fish Ingredients

The calculated ε_Trolox_ (2.62 × 10^4^ L mol^−1^ cm^−1^) in methanol/water (0.64:0.36) was used to determine the Trolox equivalent antioxidant capacity (TEAC in mmol/g) of the three commercial fish feed ingredients (A, B, and C) containing a mixture of rosemary and olive extracts. The results in increasing order of TEAC value were B (62.2 ± 2.7 mmol/g), A (68.6 ± 2.9 mmol/g), and C (71.7 ± 3.1 mmol/g). A preliminary Cochran’s C test demonstrated that the three standard deviations are comparable (*p* > 0.05). Therefore, a parametric Fisher ratio test was implemented and indicated significant differences between the three samples (*p* = 0.01). A multiple-range test, performed to detect the TEAC values that are significantly different, revealed that ingredients A and C are comparable, and significantly different from ingredient B (*p* < 0.05).

It is important to note that the TEAC measurements in this study were limited to a specific selection of fish feed ingredients, which may not fully capture the diversity of potential substrates. Future work will focus on evaluating the robustness and applicability of the method across a broader range of complex matrices, including plant-based foods, dietary supplements, and biological samples.

## 4. Conclusions

The present research is the first study reporting a validated mathematical model to select the best combination of solvents to dissolve Trolox and obtain the maximum absorption signals. The optimal solvent system, methanol and water (0.64:0.36), represents the optimal composition that allows reliable ε_Trolox_, validation parameters, and TEAC values to be determined.

Although ethanol is in the list of green solvents, its use in this study resulted in less-than-optimal assay performance. Consequently, methanol, despite not being classified as a green solvent, was chosen as a necessary compromise to achieve the desired level of analytical reliability. Given the methodological emphasis on precision and validation, this trade-off is warranted. Nonetheless, future research should prioritize the development of environmentally friendly solvent systems that offer comparable analytical performance.

Overall, the present research highlights the importance of evaluating critically and thoroughly well-established protocols before their implementation and regardless of their popularity. Unfortunately, published analytical assays for antioxidants do not describe the strategies behind the selection of specific experimental conditions, or whether they have been the result of an optimization/validation process, which may lead to confusion and conflicting results between researchers, laboratories, and publications. A decade ago, it was said that “Twenty five years of antioxidant screening have not resolved issues of assay chemistry, standardization, and reporting” [26]. As such, the field of antioxidant research has a long way to go when it comes to validation.

## Figures and Tables

**Table 1:** Summary of common antioxidant capacity assays: mechanisms, principles, detection methods, and wavelengths.

Assay	Mechanism	Principle	Analytical Method	Wavelength (nm)
ORAC	HAT	Inhibition of fluorescein (fluorescence declining)	Fluorescence	485_excitation_/520_emission_
Folin–Ciocalteu	SET	Reduction of Folin–Ciocalteu reagent (yellow → blue)	Colorimetry	765
FRAP	SET	Reduction Fe^+3^ → Fe^+2^ (orange → blue)	Colorimetry	593
CUPRAC	SET	Reduction of Cu^+2^ → Cu^+1^ (blue → orange)	Colorimetry	450
DPPH	SET + HAT	Reduction of DPPH radical to its non-radical form DPPH-H (purple → yellow)	Colorimetry	515
ABTS	SET + HAT	Quenching of ABTS radical (blue/green → colorless)	Colorimetry	734

ORAC: Oxygen Radical Antioxidant Capacity; FRAP: Ferric Reducing Antioxidant Power; CUPRAC: Cupric Reducing Antioxidant Capacity; DPPH: 2,2-Diphenyl-1-PicrylHydrazyl; ABTS: 2,2′-Azino-bis(3-ethylBenzThiazoline-6-Sulfonic acid; HAT: Hydrogen Atom Transfer; SET: Single Electron Transfer.

**Table 2:** Ternary plot diagram to guide the selection of the optimal combination of solvents that yields the maximum solubility and absorption signal (ψ) of Trolox. The $\sigma_{average}$ was used to transform the experimental signal ψ as $\Phi =\psi \;\times {\;\sigma }_{average}$ and obtain Equation (3).

Combination Number	Solvent Fraction			Experimental	Standard Deviation	
	Methanol	Ethanol	Water	Signal ψ (AU)	σ_ψ_ (AU)	$\sigma_{average}$ (AU)
1	1	0	0	1.147	0.043	0.039
	1	0	0	1.233		
	1	0	0	1.189		
2	0	1	0	0.941	0.046	
	0	1	0	1.033		
	0	1	0	0.981		
3	0	0	1	0.365	0.028	
	0	0	1	0.418		
	0	0	1	0.381		
4	0.5	0.5	0	1.201	0.064	0.046
	0.5	0.5	0	1.330		
	0.5	0.5	0	1.262		
5	0.5	0	0.5	1.237	0.044	
	0.5	0	0.5	1.324		
	0.5	0	0.5	1.266		
6	0	0.5	0.5	0.911	0.028	
	0	0.5	0.5	0.966		
	0	0.5	0.5	0.927		
7	0.33	0.33	0.33	0.933	0.060	0.060
	0.33	0.33	0.33	1.054		
	0.33	0.33	0.33	0.994		

**Table 3:** Validation of the Trolox calibration in methanol/water (0.64:0.36 v:v). The regression function was accepted as linear when both conditions, 0.900 ≤ r ≤ 1.000 and F_experimental_ < 3.2592. The LOD and LOQ were determined as 3.3 × σ_blank_/φ and 10 × σ_blank_/φ, respectively.

Trolox (μM)	Absorbance (AU)	Validation Parameters	Square Errors (AU^2^)		Accuracy (%)
			Pure	Lack-of-Fit	
0	0.033	Regression model: y = 0.013x + 0.039	1.53 × 10^−5^	7.44 × 10^−6^	0.9
0	0.039	r: 0.998	8.36 × 10^−6^	7.44 × 10^−6^	0.6
0	0.038	LOD: 0.91 μM	1.04 × 10^−6^	7.44 × 10^−6^	0.6
10	0.173	LOQ: 2.75 μM	1.13 × 10^−5^	4.90 × 10^−5^	2.8
10	0.179	Estimated ε_Trolox_: (2.62 ± 0.09) × 10^4^ L mol^−1^ cm^−1^	6.04 × 10^−6^	4.90 × 10^−5^	7.3
10	0.177	Calibration curve 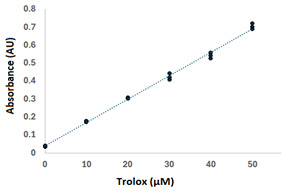	8.29 × 10^−7^	4.90 × 10^−5^	6.1
20	0.303		4.49 × 10^−6^	2.72 × 10^−5^	1.2
20	0.308		8.93 × 10^−6^	2.72 × 10^−5^	3.2
20	0.304		7.56 × 10^−7^	2.72 × 10^−5^	1.7
30	0.444		4.09 × 10^−4^	3.92 × 10^−5^	3.6
30	0.407		2.61 × 10^−4^	3.92 × 10^−5^	−5.7
30	0.419		1.64 × 10^−5^	3.92 × 10^−5^	−2.6
40	0.558		2.42 × 10^−4^	3.06 × 10^−4^	−0.4
40	0.527		2.36 × 10^−4^	3.06 × 10^−4^	−6.3
40	0.542		3.65 × 10^−8^	3.06 × 10^−4^	−3.4
50	0.723		3.39 × 10^−4^	2.03 × 10^−4^	5.0
50	0.701		1.17 × 10^−5^	2.03 × 10^−4^	1.7
50	0.689		2.24 × 10^−4^	2.03 × 10^−4^	−0.1
		Variances→	1.53 × 10^−5^	7.44 × 10^−6^	
		Degrees of freedom→	12	4	
		F_experimental_→		3.1651	
		F_theoretical_→		3.2592	

## Data Availability

Data are contained within the article.
